# Quaternary Ammonium Dimethacrylates as an Additive in Dental Composite Resins: A Review of Their Antimicrobial, Mechanical, and Physicochemical Properties

**DOI:** 10.3390/ma18214844

**Published:** 2025-10-23

**Authors:** John Ekow Ampah-Essel, Izabela Barszczewska-Rybarek, Patryk Drejka, Grzegorz Chladek

**Affiliations:** 1 Materials Research Laboratory, Faculty of Mechanical Engineering, Silesian University of Technology, Konarskiego 18A Str., 44-100 Gliwice, Poland; john.ekow.ampah-essel@polsl.pl; 2 Department of Physical Chemistry and Technology of Polymers, Faculty of Chemistry, Silesian University of Technology, Strzody 9 Str., 44-100 Gliwice, Poland; izabela.barszczewska-rybarek@polsl.pl (I.B.-R.); patryk.drejka@polsl.pl (P.D.)

**Keywords:** restorative materials, quaternary ammonium compounds, dimethacrylate, antimicrobial activity, cytotoxicity, mechanical properties, physicochemical properties

## Abstract

Dimethacrylate-based dental materials are dominantly used in restorative procedures for their mechanical and esthetic properties. However, they lack inherent antimicrobial activity, making them susceptible to microbial colonization. This has prompted the development of quaternary ammonium dimethacrylate monomers (QADMs) as a counteractive measure. This review critically assesses the tradeoffs associated with the antimicrobial potential, cytocompatibility, and structural performance of QADMs in the past decade. Across the standardized biological assays studied, QADMs consistently exhibit potent antimicrobial activity against cariogenic and opportunistic pathogens without inducing resistance. QADMs maintain favorable mechanical and physicochemical properties upon incorporation into resin composite formulations. Cytotoxicity is structure- and dose-dependent; nonetheless, most QADMs are biocompatible at antimicrobial concentrations. Notably, quaternary ammonium urethane dimethacrylate monomers (QAUDMAs) offer a balanced combination of antimicrobial and structural properties. Few studies have assessed the long-term mechanical durability of QADM-enhanced composites, leaving clinical relevance inconclusive. Further research is necessary to optimize monomer design and clinically validate these materials.

## 1. Introduction

Dental composite restorative materials based on a matrix polymerized of monomers with methacrylate groups are dominant in dentistry due to their favorable mechanical and esthetic properties [1,2]. The matrix of these materials can be composed of many types of monomers, especially bisphenol A glycerolate methacrylate (Bis-GMA), Bis-EMA, which is the ethoxylated variant of Bis-GMA, 2-hydroxyethyl methacrylate (HEMA), urethane and triethylene glycol dimethacrylates, namely UDMA and TEGDMA, respectively [3]. Approximately 75% of dental resin composites are currently based on bisphenol A glycerolate methacrylate (Bis-GMA), while 33% are based on Bis-GMA and UDMA compositions [4]. Bis-GMA exhibits low polymerization shrinkage, a high modulus of elasticity, and superior adhesion to tooth enamel, in comparison with other dental monomers; however, it is characterized by relatively high viscosity [5], leading to the need to introduce diluting, low-viscosity monomers such as TEGDMA into the system [6]. In comparison with Bis-GMA, the composition of Bis-GMA and TEGDMA shows a higher degree of conversion, which is desirable for mechanical properties, higher polymerization shrinkage, and water sorption [7,8,9]. Dimethacrylate monomers, fundamental in the formulation of dental composite matrices, are distinguished by the presence of two alkene functional groups, specifically vinyl groups. These structural features facilitate the chain-growth radical polymerization and cross-linking, enabling the formation of a robust and durable polymer network essential for dental applications [10]. During the process of polymerization, there is the formation of covalent bonds and a reduction in intermolecular spaces [11], leading to a material density increase and overall volume decrease, resulting in polymerization shrinkage and marginal gap formation [12,13], which is recognized as one of the main problems associated with the use of composites in dental treatment. Dental composite surfaces show a strong tendency to accumulate dental plaque compared with tooth tissues [10,14], which intensifies over time due to the increase in restoration surface roughness [15]. Cariogenic bacteria, such as streptococci and lactobacilli, produce acid by-products due to the metabolism of carbohydrates, which causes the demineralization of tooth tissues and triggers secondary caries following restorative procedures, ultimately undermining the longevity and success of dental restorations [16,17,18,19].

This problem emanates from the fact that dental composite restorative materials based on dimethacrylates do not possess antibacterial activity, so prior research focused on imbuing these materials with antimicrobial agents such as silver [20,21], chitosan nanoparticles [22], zinc oxide nanoparticles [23,24], calcium fluoride [25], polyethyleneimine (PEI) particles [26], chlorhexidine, and antibiotics [19,27,28]. However, these developments mostly result in short-term antibacterial activity [21,29], the elution of nanoparticles, and a decrease in performance [30,31,32], and the ability of bacteria to mutate and resist antibiotic activity, which is becoming a global health problem [33,34,35]. This persistent challenge has spurred extensive research into the integration of antimicrobial functionalities within the polymer network.

In the past decade, there has been a shift towards the development of antimicrobial dimethacrylates—novel monomer systems where quaternary ammonium groups are chemically bound to the dimethacrylate molecule, which is later incorporated into a copolymer network with methacrylate primary chains. This approach contrasts with traditional strategies that physically incorporate antimicrobial agents into the resin matrix. Antimicrobial dimethacrylates ensure that the antibacterial groups are covalently anchored within the polymer network, thereby providing sustained antibacterial activity without compromising the material’s structural integrity [36]. The evolution of antimicrobial dimethacrylates has been driven by extensive investigations into structure–property relationships. Studies have explored the influence of various factors such as N-alkyl substituent length, degree of conversion, and overall chemical structure on the antibacterial, mechanical, and physicochemical properties of the cured matrix and resulting composites. Recent studies have shown that increased alkyl chain length enhances hydrophobic interactions with bacterial cell membranes, thereby improving antimicrobial activity. However, such modifications must be carefully balanced, as overly long chains can influence polymerization kinetics, which is directly related to the degree of vinyl conversion (DVC)—further influencing the degree of cross-linking, which affects polymerization shrinkage, water sorption, water solubility, and mechanical properties—all of which are critical parameters that dictate the longevity, cytotoxicity, and clinical performance of the composite material [37]. It is worth noting that these innovative monomer systems are being designed not only to provide robust antibacterial properties but also to maintain and improve key mechanical properties. The integration of quaternary ammonium functionalities has been shown to reduce biofilm formation and diminish the occurrence of secondary caries. This is achieved via the suppression of cariogenic bacterial colonization, such as *Streptococcus mutans* and Lactobacillus species, at the tooth restoration interface. The chemically bonded antibacterial agents remain effective over prolonged periods, mitigating one of the primary drawbacks of conventional composites [38].

The novelty of this review lies in its comprehensive evaluation of antimicrobial dimethacrylates, extending beyond their antimicrobial activity to include a detailed assessment of their mechanical properties, cytotoxicity, and physicochemical behavior. Unlike previous studies that primarily focus on antimicrobial activity, this review provides a holistic analysis of these materials, identifying key research gaps essential for the development of next-generation dental restoration materials that combine durability, safety, and sustained antimicrobial performance.

## 2. Classification of Quaternary Ammonium Compounds

**Figure 1:**
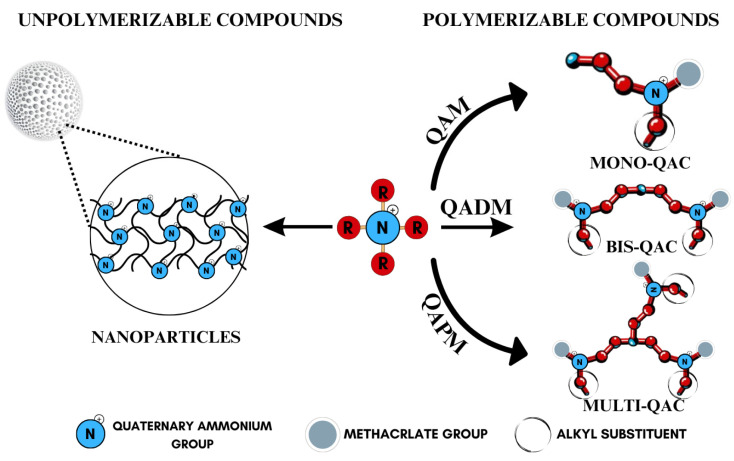
General structure and classification of quaternary ammonium compounds.

Quaternary ammonium compounds (QACs) constitute a broad category of chemicals possessing antimicrobial properties, preservation capabilities, and antistatic effects, used across various industries. Their usage significantly increased due to the COVID-19 pandemic and the 2016 U.S. FDA ban on 19 other antimicrobials in distinct personal care products [39,40,41]. Studies validated that 216 quaternary ammonium compounds were included in the U.S. Environmental Protection Agency’s List ‘N’ of approved products for effectively inactivating the SARS-CoV-2 (COVID-19) virus [42,43]. QACs are structurally characterized by a positively charged nitrogen atom bonded to four substituent groups (R_4_N^+^), enabling them to disrupt bacterial cell membranes by lowering surface tension and facilitating the efflux of intracellular components, ultimately leading to cell death [44]. These cationic compounds generally feature at least one hydrophobic substituent for antimicrobial activity [44,45]. Depending on their structural complexity, QACs are classified as having one (mono-QACs), two (bis-QACs), or many quaternary nitrogen atoms (poly-QACs), with the number of quaternary nitrogen centers influencing their antimicrobial efficiency together with the incorporation of long hydrocarbon substituent chains (Figure 1) and strategic spacer groups, which modulate their functional properties, enhancing their application in various biomedical and industrial fields.

Currently, QACs containing methacrylate groups are at the center of attention in the development of advanced dental materials, as they provide polymerizable functionality necessary for long-term structural integrity and antimicrobial efficacy. Among these, quaternary ammonium dimethacrylate monomers (QADMs) stand out as particularly promising, combining the benefits of cross-linking methacrylate groups with intrinsic antimicrobial properties. QADMs offer sustained protection against bacterial colonization, addressing the issue of secondary caries [46,47,48,49]. QADMs exhibit a dual functionality: they contribute to the structural integrity of the dental composite while simultaneously providing antimicrobial activity, which is crucial for maintaining oral health [50,51]. The presence of two double bonds reduces leakage and shrinkage as a result of polymerization from the dimethacrylate ends, resulting in potent mechanical and antimicrobial performance. The use of quaternary ammonium dimethacrylate monomers allows for the development of dental materials that can withstand the harsh oral environment, thereby extending the longevity of restorations and reducing the frequency of replacements [52], representing a significant advancement in materials science and addressing both mechanical and biological challenges inherent in dental restorations.

The impending 2025 EU ban on mercury-containing dental amalgams [53] and the growing problem of bacterial resistance are expected to drive a significant shift towards alternative restorative materials, with a notable increase in the use of quaternary ammonium-based composites. Given their dual functionality, QADMs are well positioned to replace traditional materials in dental applications as regulatory restrictions phase them out.

## 3. Quaternary Ammonium Dimethacrylate Monomers

A comprehensive bibliographic search was conducted across the PubMed and Web of Science databases, utilizing quaternary ammonium dimethacrylate as the search descriptor due to the limited but growing availability of studies. The search encompassed all study designs and covered the period from 2016 to 2025 to ensure a thorough evaluation of recent advancements. These monomers include derivatives with variations in alkyl chain length, methacrylate substitution, and spacer architecture, each influencing antimicrobial activity, hydrophobicity, compatibility, and polymer network integration.

**Figure 2:**
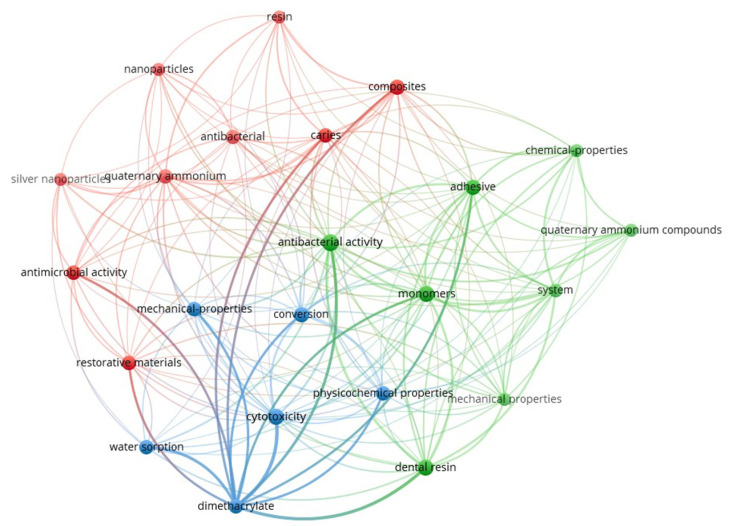
Keyword co-occurrence network on quaternary ammonium dimethacrylate from literature (2016–2025).

Figure 2 presents the keyword co-occurrence network generated using VOSviewer (version 1.6.20) [54], based on research articles retrieved during this study. The map visually highlights the thematic structure and research clusters associated with quaternary ammonium dimethacrylate monomers in the recent dental materials literature. The interconnected keywords, their frequencies, and total link strengths are provided in Supplementary Information (Supplementary Table S1).

The QADMs investigated in this review are presented in Table 1 along with their structures and analyzed properties. The reported monomers possess potential for enhancing bioactivity and mechanical stability in dental resin systems, and their synthesis and nomenclature are consistent with the prior literature.

### 3.1. Antimicrobial Activity

#### 3.1.1. Mechanism of Antimicrobial Action

The evaluated studies reveal that QADMs exert their antibacterial effects through a multi-step contact-active mechanism, integrating both physical disruption and biochemical interference. These mechanisms stem primarily from the quaternary ammonium moieties integrated into the monomer structure and, further, the cross-linked polymer matrix.

**Figure 3:**
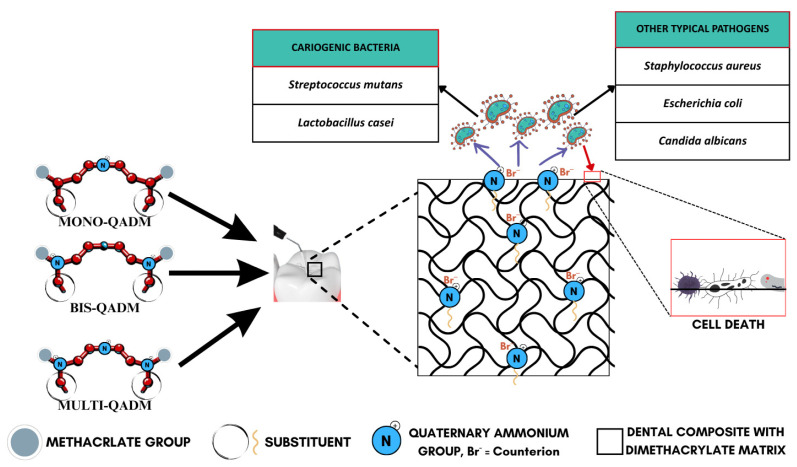
Antimicrobial action of quaternary ammonium dimethacrylate monomers.

**Figure 4:**
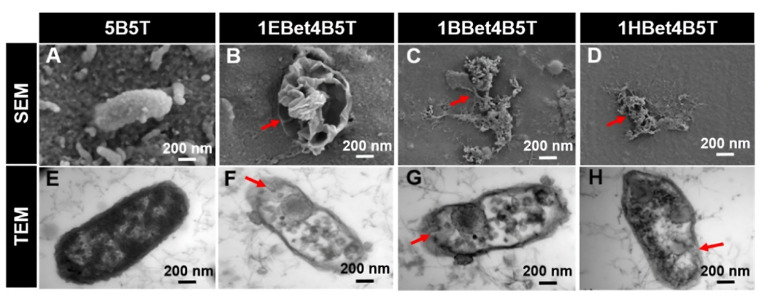
(**A**–**D**) SEM and (**E**–**H**) TEM images illustrating membrane damage and cytoplasmic leakage of *S. mutans* after 24 h incubation. Red arrows highlight changes in damage with varying alkyl chain length. Reproduced with permission [59].

The foundational mechanism is electrostatic attraction, where the positively charged quaternary groups (R_4_N^+^) on the surface interact with the negatively charged bacterial cell wall, as illustrated in Figure 3, effectively destabilizing the electrical equilibrium of the bacterial membrane [59,60,64,66]. This interaction eventually causes the microbial membrane to rupture, leading to cytoplasmic leakage and cell death (Figure 4). This is supported by consistent evidence from studies showing durable antimicrobial activity, even after long-term water aging [66], suggesting minimal monomer leaching and sustained surface-bound activity. The antimicrobial efficacy is further amplified by the hydrophobic alkyl chains attached to the quaternary nitrogen. These chains are hypothesized to penetrate the lipid bilayer of the bacterial membrane, causing physical disruption and leakage of essential ions such as K^+^, Fe^3+^, and Mg^2+^, as well as proteins [59]. The length and structure of these alkyl chains are crucial, as medium-length alkyl chains have shown optimal activity while too-short or too-long chains reduce efficiency. This membrane-targeting effect is a distinctive advantage of QADMs over traditional non-polymerizable antimicrobials.

QADMs also exhibit a unique biochemical mechanism by inhibiting gene expression associated with biofilm formation, specifically targeting *Streptococcus mutans* glucosyltransferase genes (*gtfB*, *gtfC*, and *gtfD*) [59,70]. These enzymes synthesize water-insoluble glucans, which are the main components of extracellular polysaccharides in the dental biofilm, facilitating bacterial adhesion and biofilm development. The suppression of these genes leads to disrupted biofilm metabolism, further reinforcing microbial elimination and preventing secondary caries. Compared to monomethacrylate quaternary ammonium monomers, QADMs exhibit higher surface charge density [60], as well as reduced monomer leachability due to effective incorporation into a polymer network. This structural advantage enhances antimicrobial durability and mechanical compatibility with conventional dimethacrylates like Bis-GMA and TEGDMA. The role of the quaternary ammonium counterion remains ambiguous. Some studies suggest variations in antibacterial activity depending on the counterion type, while others find no significant effect [60,71].

#### 3.1.2. Antimicrobial Properties

The antibacterial activity of QADMs has been investigated using multiple standardized assays. Across all studies reviewed, its antimicrobial potential has been assessed through Minimum Inhibitory Concentration (MIC)/Minimum Bactericidal Concentration (MBC), Inhibition Zone Diameter (IZD), bacterial/fungal adhesion, a live/dead biofilm staining assay, gene expression analysis, protein leakage and ion-release analysis, the lactate dehydrogenase-based enzymatic method to measure lactic acid production, and the 3-[4,5-dimethylthiazol-2-yl]-2,5-diphenyltetrazolium bromide (MTT) assay to examine the metabolic activity of biofilms [49,50,51,56,59,60,61,62,63,64,66,69,70]. While comparisons between studies must be approached with caution due to singular use or monomer concentration, experimental formulations, and bacteria species tested, the collective findings consistently demonstrate QADMs’ superior antimicrobial performance relative to the control specimen. Specimen tested include (i) Gram-positive bacteria: *Streptococcus mutans*, *Streptococcus sanguinis*, *Streptococcus mitis*, *Staphylococcus aureus*, *Lactobacillus casei*, and *Bacillus subtilis,* and (ii) Gram-negative bacteria: *Escherichia coli*, *Pseudomonas aeruginosa*, and (iii) the fungus *Candida albicans*.

*Streptococci*, particularly *Streptococcus mutans*, are widely recognized as primary contributors to dental caries due to their acidogenic nature. They metabolize sugars into acids and create a low-pH environment that demineralizes the enamel and compromises restorative materials. Given their central role in caries development, the antimicrobial activity of QADMs against these pathogens has been the focal point in evaluating their potential. A recurring theme across published studies is the dependence of antibacterial activity on the monomer structure and incorporation method. Zhang et al. [59] synthesized betulin-based bis-QADMs with varying alkyl chains (EBet-C4, BBet-C8, HBet-C12). When added to Bis-GMA/TEGDMA resin at 10 wt.% (1EBet4B5T, 1BBet4B5T, and 1HBet4B5T), these monomers achieved a >99% reduction in S.mutans colonies, with the C8 derivative proving most effective. Protein leakage and TEM imaging confirmed disruption of bacterial membranes, while gene expression assays showed the downregulation of *gtfB* and *gtfC*, weakening biofilm formation. Cytoplasm leakage of bacterial extracellular protein observed in TEM images was 0.2 mgmL^−1^ for the control, with 1EBet4B5T, 1BBet4B5T, and 1HBet4B5T recording significant leakage increases of 0.6, 0.7, and 0.6 mgmL^−1^, respectively. Similarly, MAE-HB incorporated at 10% into Bis-GMA/TEGDMA resin showed reduced *gtfB* and *gtfC* activity, though *gtfD* was unaffected [70].

Other investigations, such as by Fanfoni et al. [60], compared broad families of dimethacrylate monomers—DDM, DDM-F, and DDMP, DDMP-F, DDE, DDMAPMA, DDPyMMA, XyDM, and BPDE—against commercial references like MDPB. Derivatives such as DDPyMMA and DDMAPMA showed higher activity with very low MIC/MBC values, while others were only moderately effective or inactive. The observed MIC and MBC results show that BPDE (MIC = 2500 µg/mL, MBC = 10,000 µg/mL) and XyDM (MIC = 2500 µg/mL, MBC = 2500 µg/mL) were less active against *S. mutans* at a similar magnitude to IDMA-2 and a thousand times more active compared to MDPB. Similar MIC and MBC values were observed for DDM (MIC = 150 µg/mL, MBC = 300 µg/mL), DDM-F (MIC = 156 µg/mL, MBC = 313 µg/mL), DDMP (MIC = 150 µg/mL, MBC = 310 µg/mL), and DDMP-F (MIC = 156 µg/mL, MBC = 156 µg/mL), demonstrating intermediate activity against *S. mutans* as compared to the other monomers. DDPyMMA showed higher inhibitory and bactericidal activity (MIC = 2.5 µg/mL, MBC = 2.5 µg/mL) than MDPB (MIC = 4.0–8.0 µg/mL, MBC = 8 µg/mL), followed by DDMAPMA (MIC = 5 µg/mL, MBC = 10 µg/mL) and DDE (MIC = 10–20 µg/mL, MBC = 20 µg/mL). Importantly, many of these findings were based on unpolymerized monomers tested in solution.

By contrast, studies on polymerized systems often report lower activity but emphasize contact-dependent killing, which is a mechanism poorly captured by solution-phase methods. Solution-based assays emphasize leaching activity rather than performance under polymerized, non-diffusible conditions. Huang et al. [68] provided a clear example of this methodological issue by synthesizing IMQ-16 and incorporating it into a UDMA/SR833s resin. While direct contact testing against *S. mutans* revealed significant bacteria reduction at 17–20% incorporation (3.91–4.38 LgCFU), the agar diffusion test showed no inhibition zones. This confirmed that the antibacterial action was contact-dependent rather than reliant on leaching. Manouchechri et al. [62] demonstrated strong antibacterial effects when DMBH and DMBB were incorporated into Tetric N-Bond, with MIC testing of the uncured sample in solution suggesting high potency. The commercial adhesive Tetric N-Bond, without any antibacterial component, was used as the control. Average MIC for the control was 2.4 × 10^3^ µg/mL. The control + DMBH exhibited a MIC of 3.12 µg/mL, with the control + DMBB exhibiting a MIC of 6.25 µg/mL. The inhibition zone diameter (IZD), on the other hand, provided less concise insight given the cured, non-leaching nature of the material. The highest IZD (9 mm) was obtained for the control + DMBH, followed by control + DMBB (7 mm), and the control (2 mm). Wang et al. [63] found DPHB and DPNB inhibited *S. mutans* biofilms above 10–5 M concentrations, while DPDB was inactive, underscoring the significant influence of alkyl chain variation on biological outcomes.

The contrast between these methodologies is evident in several studies. Li et al. [61] observed a dose-dependent antibacterial effect when their synthesized bio-based QANMA monomer was incorporated into Bis-GMA/TEGDMA resin at 5–20 wt.%, but this was primarily assessed through direct bacterial contact rather than diffusion-based assays. 5 wt.% QANMA incorporation showed no significant difference from the control. The results of the bacteria LIVE/DEAD staining assay of *S. mutans* were consistent with the bacteria colony counting assay and showed a trend of decreasing live bacteria with increase in QANMA concentration in the resin system. Noticeably, 20% QANMA incorporated into the resin system had the highest dead bacteria. Similarly, Makvandi et al. [69] developed QABGMA and incorporated it into Bis-GMA/TEGDMA at 5–15 wt.%. The results showed concentration-dependent inhibition of *S. mutans*, with higher activity than conventional antibiotics such as gentamicin and chloramphenicol.

When composites are examined under more clinically relevant conditions, results are mixed but instructive. Huang et al. [64] reported suppression of *S. mutans* by UDMQA-12-containing resin comparable to that of glass ionomer cement, though bacterial regrowth was evident within 48 h. Cheng et al. [66] extended this by formulating composites with IDMA-1, silver nanoparticles, and amorphous calcium phosphate. Their systems maintained antibacterial activity for up to 12 months of water aging, with consistently reduced biofilm viability and lactic acid production. Such durability is promising, especially given that contact-active systems must demonstrate long-term performance without relying on leaching.

Beyond *S. mutans*, investigations into other species provide a broader picture of QADM activity. *Lactobacillus casei*, a Gram-positive acidogenic bacterium, contributes to lesion progression once streptococci initiate caries. A growth inhibition assay in MRS medium was used by Wang et al. to evaluate *Lactobacillus casei* proliferation based on optical density measurements on synthesized monomers DPDB, DPHB, and DPNB [63]. Monomers DPHB and DPNB exhibited inhibitory activity at 10^−5^ M concentrations. DPDB, however, exhibited the strongest inhibitory activity against *L. casei* at 10^−4^ M concentrations. *Staphylococcus aureus* has served both as an opportunistic and biomaterial-relevant strain. QAUDMA derivatives with varying alkyl chain lengths incorporated into Bis-GMA/TEGDMA resin systems completely prevented colony formation in some formulations, while others exhibited MIC/MBC ranging from 6.25 to >50 mg/mL, with changes due to increasing alkyl chain length of the quaternary ammonium compound. Other bacteria such as *Escherichia coli*, *Pseudomonas aeruginosa*, and *Bacillus subtilis* have also been studied [63,69]. *Escherichia coli,* especially, has been used as a general test strain in antibacterial studies, though not a typical oral bacterium. *E. coli* is most often used as a model reference point for comparing activity and monomer efficacy across gram classes. Activity against *E. coli* ranged from 2.5 µg/mL inhibitory concentrations for DDPyMMA and DDMAPMA to 156 µg/mL for less active derivatives such as DDE [50,60]. Adhesion studies further demonstrated that the QADM-incorporated resin could reduce *E. coli* surface colonization by several counts, and in some cases to non-detectable levels.

*Candida albicans*, the primary fungal agent of oral candidiasis, has also been evaluated [59,72,73]. Fungal adhesion was suppressed with inhibition zones between 7 and 13 mm, particularly when the alkyl chain length was below C16 [51]. QABGMA showed strong antifungal activity even at concentrations lower than commercial antibiotics [69]. This extension to fungal pathogens is highly relevant to immunocompromised patients as it underscores the potential of QADMs to act not only against bacterial biofilms but also mixed microbiomes.

The available evidence indicated that QADMs exhibit broad-spectrum antimicrobial activity shaped by monomer design, concentration, and mode of incorporation. Solution-based assays reveal intrinsic potency, but polymerized systems provide a more realistic assessment of long-term, contact-active performance. A consolidated table summarizing key findings across the microbial strains is provided in the Supplementary Information (Supplementary Table S2).

### 3.2. Cytotoxic Effect

Studies assessing the cytotoxicity of quaternary ammonium dimethacrylate (QADM) monomers used standardized protocols (ISO 10993-5 [74]) but employed different cell lines and viability assays.

Chrószcz-Porębska et al. [51] used L929 mouse fibroblast cells and the Alamar Blue assay to evaluate BG:QAm:TEG copolymers. The cell viability ranged from 63.81% to 76.77%, with BG:QA8:TEG showing significantly lower viability compared to copolymers with QA12 and QA18, and all BG:QAm:TEG copolymers exhibiting reduced viability relative to the control (BG:UD:TEG). Zhang et al. [59] investigated human dental pulp cells (HDPCs) using the CCK-8 assay and Calcein-AM/PI staining over 1–7 days. Results indicated sustained cell proliferation and high viability for experimental resins (1EBet4B5T, 1BBet4B5T, and 1HBet4B5T), even exceeding the control (5B5T) resin after 3 days, suggesting minimal cytotoxic effects. Fanfoni et al. [60] utilized human dental pulp stem cells (HDPSCs) to assess monomers DDM, DDE, DDMAPMA, and DDPyMMA via 3-(4,5-dimethylthiazol-2-yl)-5-(3-carboxymethoxyphenyl)-2-(4-sulfophenyl)-2H-tetrazolium (MTS) growth assay over 24 and 72 h. A dose-dependent response was observed, with DDM tolerable up to 2 mg/mL, while the other monomers were in the range of 50–100 µg/mL. Notably, these concentrations exceeded their respective minimum inhibitory and bactericidal thresholds, indicating a favorable safety profile. Cytotoxicity was evaluated on human foreskin fibroblast (HFF2) by Manouchechri et al. [62] using MTT assay on polymerized disks with DMBB and DMBH. They found that while both showed some toxicity, cured resins containing 1 wt.% DMBB maintained higher cell viability than the commercial adhesive, whereas 1 wt.% DMBH showed reduced viability, suggesting the need to balance antibacterial efficacy and biocompatibility. Wang et al. [63] used human gingival fibroblasts treated with different concentrations (10^−4^–10^−7^ M) of their novel monomers, and analyzed cell survival via Calcein-AM fluorescence, revealing that the higher toxicity was observed with C16 aliphatic chain monomers at 10^−4^ M, while shorter C11 chain monomers demonstrated lower cytotoxicity than Bis-GMA, highlighting the impact of hydrophobic chain length on cell compatibility. Huang et al. [64] evaluated cytotoxicity using HDPCs and the CCK-8 assay, comparing the experimental composite resin with conventional composites and glass ionomer cement (GIC), concluding that both composite types showed low cytotoxicity and maintained normal fibroblast morphology, in contrast to GIC, which induced notable morphological changes and reduced density. Bienek et al. [65] demonstrated that exposure to IDMA-1 (≤10.66 mmol/L) and IDMA-2 (≤5.70 mmol/L) over 24–72 h resulted in minimal cytotoxic effects. Specifically, cell viability under IDMA-1 exposure dropped only by 5.5% after 72 h, with no statistically significant changes observed across concentrations or time for either monomer. Makvandi et al. [69] reported a marked dose-dependent cytotoxic response to QABGMA-containing resins. Resins with 5% QABGMA exhibited high fibroblast viability and negligible morphological changes, confirming good biocompatibility. However, increasing the QABGMA content to 10% and 15% led to significantly reduced absorbance values at 570 nm in MTT assays, correlating with a steep decline in viable cell numbers.

### 3.3. Mechanical and Physicochemical Properties

The mechanical and physicochemical performance of quaternary ammonium dimethacrylate monomers (QADMs) incorporated into copolymers or composite matrices is summarized in Table 2 below. Mechanical properties investigated include hardness (HV), elastic modulus (E), flexural strength (FS), and compressive strength (CS), while physicochemical properties encompass water sorption (SL), water solubility (SL), degree of conversion (DC), polymerization shrinkage (S_e_, S_T_), glass transition temperature (T_g_), and water contact angle (WCA). Extracted data assessed the physical properties of QADM-modified compositions under standardized testing conditions, highlighting the influence of QADM content and molecular architecture on both the structural integrity and functional behavior of dental materials.

The mechanical and physicochemical properties of QADM-modified resins and composites have been evaluated against conventional dimethacrylate formulations to determine their suitability for clinical applications. Standardized benchmarks such as those outlined in ISO 4049 [75] for polymer-based restorative materials serve as key references when assessing these systems, which stipulate a minimum flexural strength of 80 MPa, as well as standard limits on water sorption (≤40 μg/mm^3^) and solubility (≤7.5 μg/mm^3^).

Most studies report that QADM-modified resin copolymers meet the dental standards, though a few outliers fail on water uptake and degree of conversion. In practice, they mostly meet the solubility limit with copolymers showing SL ~2.18–6.61 μg/mm^3^. Water sorption varies with alkyl chain length and compositional addition of QADM. Longer-chain resins (C12–C18) all had WS typically ~10–35 μg/mm^3^, while shorter chains exceeded it, reaching ~138 μg/mm^3^. Notably, betulin-based QADMs had DC < 50%. In other words, except for very hydrophilic systems, these QADM systems comply with ISO moisture-uptake criteria. In contrast, their mechanical strengths usually fall short of ISO 4049’s recommendation. For instance, QAUDMA/TEGDMA (60:40 wt.%) had a hardness of ~41–51 MPa and flexural strength of ~20–37 MPa, which is roughly half the hardness and lowest in strength compared to the Bis-GMA/TEGDMA resin in the same test. This is common for most of the copolymers, as some authors explicitly note that their resins cannot replace standard Bis-GMA/TEGDMA composites due to insufficient mechanical properties [57]. Notably, a few compositions from QADMs other than QAUDMAs exceeded the reference benchmark.

From mapping the table, it is evident that QADMs usually conform to ISO 4049’s chemical criteria but typically fail their mechanical counterparts. The root cause is well documented: the quaternary groups make the network more hydrophilic and reduce crosslink density, so water uptake goes up while strength goes down [57,58]. Hence, most QA-containing copolymers exceed the WS limit as a result of being too hydrophilic or fall short of the ≥80 MPa requirement, except in the carefully optimized medium-chain cases.

## 4. Discussion

Though the quaternary ammonium dimethacrylate monomers (QADMs) in this review share at least two methacrylate (C=C double bond) functionalities to allow radical crosslinking, their divergence in spacer chemistry and quaternary nitrogen leads to distinct physical and antimicrobial properties. QAUDMA-derivatives incorporate diisocyanate-derived cores–aliphatic (QAUDMA-m), cycloaliphatic (IDP, CHMDI, UDMQA-12), or aromatic (MDI, TMXDI)–flanked by urethane-linked methacrylate arms bearing two cationic centers; resulting in a bisquaternary architecture with strong cross-linking properties. Betulin-derived QADMs incorporate a bulky pentacyclic triterpenoid core with side chains bearing quaternary ammonium (QA)-functionalized DMAEMA; with varying alkyl chains such as ethyl (EBet), butyl (BBet), or hexyl (HBet). DDM and DDM-F, along with their amide-based analogues DDMP and DDMP-F, represent bis-quaternary dimethacrylates with dodecyl QA chains, and ethyl or amino-propyl linkers, with differing halide counterions (Br^−^ or F^−^). This may modulate ionic dissociation and surface charge while maintaining an entirely aliphatic nature. Similarly, DDE, DMBB, and DMBH are bis-QADMs with tetraethyl or tetramethyl QA heads and dodecyl, butanediyl, and hexanediyl alkyl spacers, respectively, varying in flexibility and intermolecular spacing. DDMAPMA and DDPyMMA incorporate para-substituted phenylene and pyridinium rings, respectively, providing added rigidity and most likely π-conjugation, which may influence surface interactions and electronic stability. XyDM and BPDE similarly incorporate xylyl and benzyl-based central spacers, contributing moderate aromaticity and structured packing. This is particularly important for stability in biological systems. Pyridinium-containing QANMA with a hexyl carbonyl linker and a single aromatic ring combines moderate rigidity with hydrophobicity, while MAE-HB contains a long hexadecyl QA tail, increasing mobility and surface antibacterial potential, though also raising concerns about diffusion and leaching. IDMA-1 and IDMA-2 mainly differ by their aliphatic and aromatic bridges, respectively, which contribute to their stiffness and potential charge distribution. DPDB, DPHB, and DPNB incorporate long aliphatic linkers esterified to dimethacryloyloxypropyl QA moieties, with azabicyclooctyl (DPDB, DPHB) or benzyl (DPNB) substituents, designed for deep membrane disruption and potent antibacterial function. Similarly, IMQ-16 is an amide containing QADM, featuring a central hexadecyl-substituted core, optimizing hydrophobic contact and mechanical integration. Contrary to the UDMA-derivatives, QABGMA leverages the rigidity of bisphenol A glycerolate functionalized with QA and dimethacrylate terminals, highlighting the versatility of Bis-GMA, possessing a rigid network coupled with a potentially sustained antimicrobial activity. Across all monomers, the balance between aliphatic and aromatic moieties, chain length of QA substituents, spacer flexibility, and the number of reactive sites influences each monomer’s ability to integrate into polymer matrices, resist leaching, maintain mechanical strength, and deliver broad-spectrum antimicrobial activity.

**Figure 5:**
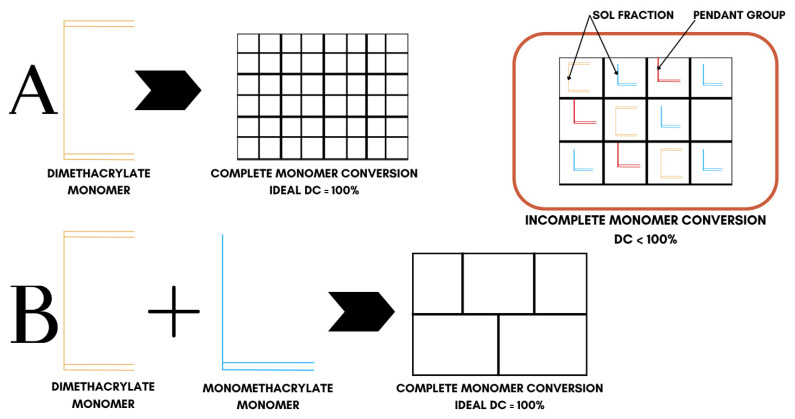
Conversion behavior in crosslinked dimethacrylate networks. System ‘A’ represents ideal conversion behavior of dimethacrylate monomers while system ‘B’ represents the ideal conversion behavior of dimethacrylate and monomethacrylate monomer mixtures.

In all cases, polymerization produces highly heterogeneous networks, because the ‘ideal network’ model, which is complete monomer conversion, is never realized. As illustrated in Figure 5, real dimethacrylate networks contain pendant groups, loops, and microgel clusters; thus, double-bond conversion (DC) never reaches 100% [76] (Table 2), displaying a heterogeneous conversion behavior. Bulky QADM structures increase autoacceleration, trapping unreacted C=C bonds. For this reason, there is a necessity to balance monomer design against crosslinking efficiency and network integrity. Among the QADMs, a clear pattern is observed: increasing the QA moieties and alkyl chain length generally may improve antimicrobial potency through enhanced membrane disruption, but at the cost of higher hydrophilicity and lower crosslink density. While the QA headgroups are inherently hydrophilic, the attached alkyl chains counterbalance that by providing a hydrophobic character, and thus, monomers with longer alkyl spacers often display lower overall water sorption than shorter-chain analogues, provided the ionic QA content remains the same. Monomers with aromatic cores often yield lower sorption but can raise solubility [77].

Compared to conventional dimethacrylates, many QADM-modified resins exhibit an altered cure behavior. For instance, most of QAUDMA resins show increasing polymerization conversion with increasing alkyl chain length, often exceeding that of Bis-GMA/TEGDMA reference polymers. Their copolymers with Bis-GMA/TEGDMA exhibit markedly low volumetric shrinkage and moderate-to-high glass transition temperatures. Mechanical testing on QAUDMA-based copolymers showed flexural strength mostly between 50 and 100 MPa and modulus between 2 and 3.7 GPa, so they are comparable to conventional Bis-GMA/TEGDMA and UDMA/TEGDMA controls. Notably, the mid-range N-alkyl chain lengths (C8-C12) produced the best mechanical performance while also imparting high antibacterial activity. In liquid formulations, QAUDMAs also show higher glass-transition and viscosity than Bis-GMA, reflecting their bulky structure. They are typically liquid at room temperature, but very viscous, requiring diluents such as TEGDMA to form workable resins. Chrószcz et al. [58] reported that copolymers 60 wt.% QAUDMA with varied alkyl chains, and 40 wt.% TEGDMA had distinct trends: hardness decreased as the N-alkyl chain lengthened, while flexural strength and modulus increased up to C10 and then declined. These mechanical changes paralleled viscosity as short-chain monomers yielded stronger networks. However, none of the QAUDMA:TEGDMA copolymers matched the strength of a Bis-GMA/TEGDMA control.

In contrast, betulin-based resins at 10 wt.% with Bis-GMA/TEGDMA achieved above 99.9% *S. mutans* reduction without sacrificing basic mechanical properties. At 10 wt.% addition, BBet and HBet gave flexural strengths comparable to the unmodified control, while EBet lowered strength significantly. The resin conversion was lower even at 10 wt.% as compared to QAUDMAs and the Bis-GMA/TEGDMA reference. Contact angles showed that longer alkyl chain lengths, such as for HBET, made the surface more hydrophobic. Dimethacrylate analogues bearing longer alkyl quaternary amines, such as DDM, DDE, DDMMP, and DDMAPMA, are generally characterized by higher molecular weight. Their incorporation tends to raise resin viscosity only slightly (depending on the spacer flexibility), moderately increasing conversion, but may reduce crosslink density if only one acrylate reacts. For instance, IDMA-1 was fully miscible with Bis-GMA/TEGDMA and raised the degree of conversion with no significant resin shrinkage penalty. However, higher concentrations of mono-QADMs such as exceeding 20 wt.% can reduce flexural strength and increase water sorption [61], so in practice, most studies use 5–15 wt.% of these QADMs to balance properties [68,70]. Alternatively, multifunctional crosslinkers like tetrafunctional TMQA enhance network rigidity [78,79].

QADMs are mostly polymerized into the resin matrix to yield a non-leaching contact-killing surface. In vitro tests show that nearly all the above-mentioned monomers confer strong antibacterial action against caries-associated bacteria. Generally, bis-QADMs outperform mono-QADMs at equal content, with most showing low cytotoxicity at concentrations needed to deliver the antibacterial action. However, in practice, relatively few studies measure cytotoxicity alongside antibacterial activity. This makes cross-study comparisons difficult as antibacterial assays vary widely, and cytotoxicity tests use different cell types and endpoints. Any apparent trade-offs are hard to generalize across disparate reports.

Some studies have nevertheless evaluated both aspects. These studies demonstrated dramatic suppression of *S. mutans* compared to control, with 25 mg/mL leaving no viable colonies of *S. aureus* or *E. coli* on agar [51,64,65]. At the same time, L929 fibroblast viability remained high, showing only a 12–27% reduction in cell viability compared to control. In direct-contact tests, the QADM resin completely inhibited bacterial growth, yet the pulpal fibroblast viability was statistically identical to a conventional composite (*p* > 0.05). Under ISO 10993-5, which outlines a >30% reduction to be cytotoxic, these QADMs qualified as non-cytotoxic. These isolated case studies suggest a broad pattern: QADMs can achieve strong microbial killing at doses that only modestly reduce mammalian cell viability. Mapping such results onto ISO 10993-5 criteria helps clarify the picture. In fact, the QAUDMA copolymers were explicitly deemed non-cytotoxic by ISO standard. Nevertheless, the diversity of methods and endpoints makes cross-comparisons unreliable as one cell line may be much more sensitive than the other, and the polymer network can differ greatly. It is therefore difficult to say universally QADM ‘X’ is better than ‘Y’. Each copolymer system must be judged on its own antimicrobial-toxicity balance. It bears emphasizing that meeting the ISO standard is not the ultimate goal. Ideally, we want as high viability as possible.

No study to date has directly addressed whether nitrogen-center chirality in a QADM alters its antimicrobial behavior. In fact, most dental QADMs have two identical substituents on nitrogen; thus, they are achiral, and this question does not arise. However, related work on chiral quaternary ammonium salts suggests that stereochemistry can matter. Ramić et al. [80] prepared pairs of quasi-enantiomeric quaternary salts derived from cinchonidine (CD) and cinchonine (CN) alkaloids. They found that the CD series consistently had higher antibacterial potency than the CN series. Generally, quaternary derivatives of CD showed more antibacterial activity than the corresponding quasi-enantiomer CN. This entioselectivity implies that even a single stereocenter at the quaternary nitrogen can dramatically affect how the cation interacts with microbial membranes. Specifically, chirality could influence the orientation of the positively charged head relative to the alkyl chains, potentially altering membrane disruption and binding to cell components. Investigating this remains an open challenge, and future work could make and test each enantiomer of a chiral QADM to see if handedness at the quaternary center truly affects bacterial killing, as it does in these model compounds.

Experiments regarding novel syntheses and in vitro testing of QADMs with diversity in structures have been explored, leading to a better understanding of the patterns of the relationship between chemical composition, structure, and functional properties. However, clinical studies evaluating dental composites containing QADMs have not yet been reported in the available literature. Among the reviewed studies, only a few assessed the antibacterial activity of composites incorporating QADMs [64,66], while the rest were focused on isolated monomers or copolymer formulations, offering limited insight into antibacterial, immunological, mechanical, and physicochemical performance of composites. Thus, no consistent trends or clear compositional–property relationships could be established across different QADM structures yet. The mode of antimicrobial action warrants closer scrutiny as some studies suggest monomer leaching, which may raise cytotoxicity concerns. Future work should emphasize a comprehensive evaluation of QADM-based composites, including assessments of shrinkage, water sorption, mechanical behavior, and long-term biofilm inhibition.

## 5. Conclusions

This review presents a comparative analysis of structurally diverse quaternary ammonium dimethacrylate monomers (QADMs), revealing critical insights into their physicochemical, mechanical, and antibacterial properties as they relate to dental applications. For practical selection of QADMs, different structural classes offer distinct trade-offs,; however, quaternary ammonium urethane dimethacrylate monomers (QAUDMAs) emerge as the most balanced choice across all properties and provide the best compromise of all required properties. Looking ahead, the next phase of QADMs should prioritize smarter monomer designs and rigorous real-world testing. Researchers could refine the chemical makeup of QA dimethacrylates by optimizing alkyl chain lengths, backbone rigidity, and crosslinking ability to strike the ideal balance between a tough, low-sorption polymer network and a highly active antimicrobial surface. Coincidentally, engineers should explore hybrid composite systems that pair these active monomers with remineralizing fillers so that new restoratives not only kill bacteria on contact but also help heal the adjacent tooth structure. Future work must go beyond simple lab assays by mimicking the oral environment more closely, and incorporating long-term water aging, mechanical fatigue, thermal cycling, and realistic multi-species biofilms. Through thoughtful molecular design coupled with clinically relevant testing, future research can pave the way for a new class of dental materials that unite lasting physicomechanical resilience with anti-caries performance, ensuring restorations are safe for years in a real mouth.

## Figures and Tables

**Table 1:** Evaluated quaternary ammonium dimethacrylate monomers from primary studies, with their structures and properties studied.

Monomer	Structure	Properties Analyzed	Ref.
QAn+IPDI (n = 8 or 10) m = 5 or 7	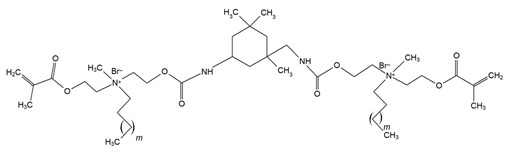	Water sorption (WS); Water solubility (SL).	[55]
QAn+CHMDI (n = 8 or 10) m = 5 or 7	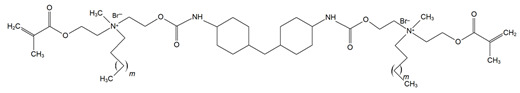		
QAn+MDI (n = 8 or 10) m = 5 or 7	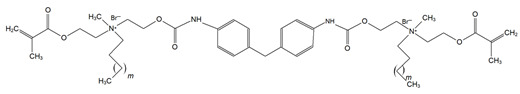		
QAn+TMXDI (n = 10 or 12) m = 9 or 11	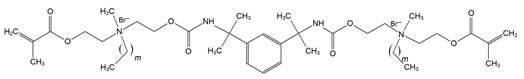	Degree of conversion (DC); Hardness (HB); Flexural modulus (E); Flexural strength (FS); Density (d_p_); Polymerization shrinkage (S_e_); Glass transition temperature (T_g_); Minimum inhibitory concentration (MIC); Minimum bactericidal concentration (MBC).	[50]
QAUDMA-m (m = n + 1, n = 7, 9, 11, 13, 15, 17)	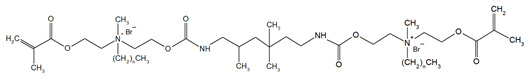	Degree of conversion (DC); Flexural strength (FS); Flexural modulus (E); Hardness (HB); Bacterial adhesion (CFU); Inhibition zone diameter (IZD); Cytotoxicity; Fungal adhesion (CFU); Glass transition temperature (T_g_); Experimental and theoretical polymerization shrinkage (S_e_, S_t_); Water contact angle (WCA); Water sorption (WS); Water solubility (SL).	[49,51,56,57,58]
Bis-QADM-Bet EBet, R_1_ = -CH_2_- BBet, R_2_ = -C_3_H_6_- HBet, R_3_ = -C_5_H_10_-	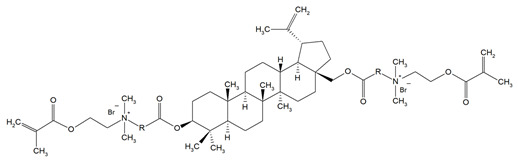	Degree of conversion (DC); Flexural strength (FS); Flexural modulus (E); Compressive strength; Cell viability; Antibacterial activity.	[59]
DDM (X^−^ = Br^−^) DDM-F (X^−^ = F^−^)	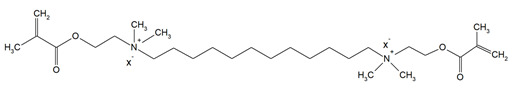	Minimum inhibitory concentration (MIC); Minimum bactericidal concentration (MBC); Cytotoxicity.	[60]
DDMP (X^−^ = Br^−^) DDMP-F (X^−^ = F^−^)	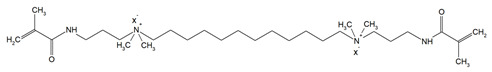		
DDE	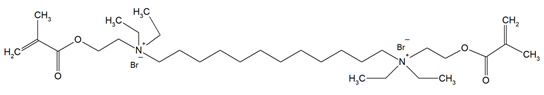		
DDMAPMA	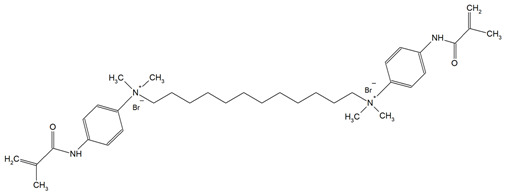		
DDPyMMA	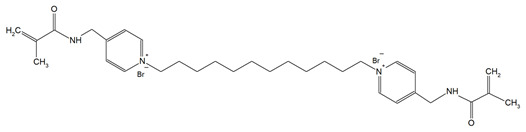		
XyDM	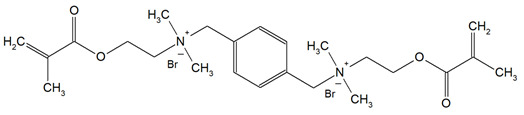		
BPDE	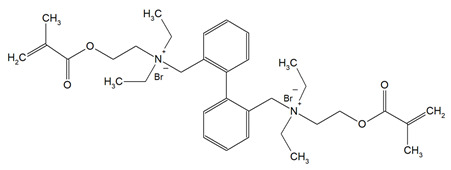		
QANMA (n = 5)	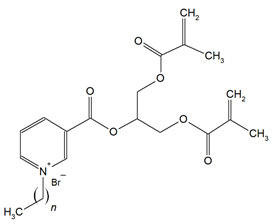	Double bond conversion (DC); Polymerization shrinkage (volumetric, S_e_); Water sorption (WS); Water solubility (SL); Flexural strength (FS); Flexural modulus (E); Antibacterial activity.	[61]
DMBB	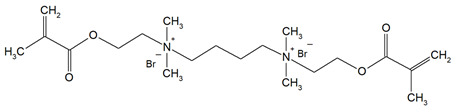	Degree of conversion (DC); Thermal stability; Bond strength (BS); Antibacterial activity.	[62]
DMBH	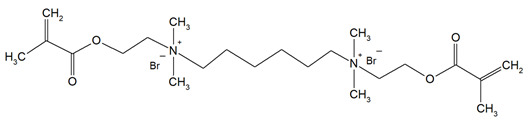		
DPDB (n = 8)	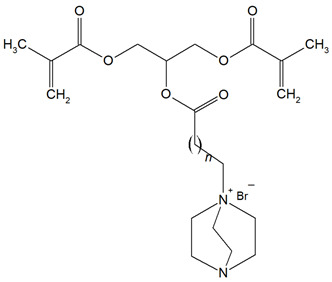	Cytotoxicity; Antibacterial activity.	[63]
DPHB (n = 14)	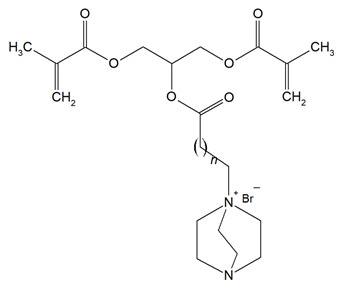		
DPNB (n = 14)	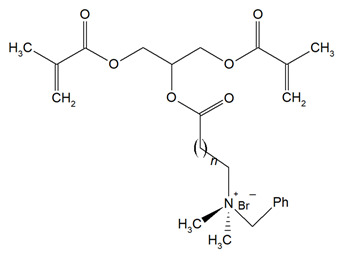		
UDMQA-12 (n = 9)	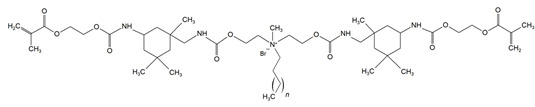	Antibacterial activity; Cytotoxicity; Flexural strength (FS); Flexural modulus (E).	[64]
IDMA-1	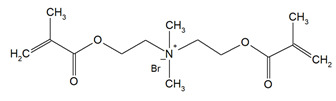	Biocompatibility; Contact angle (CA); Degree of conversion (DC); Biaxial flexural strength (BFS); Flexural strength (FS); Flexural modulus (E); Live/dead bacteria.	[65]
IDMA-2	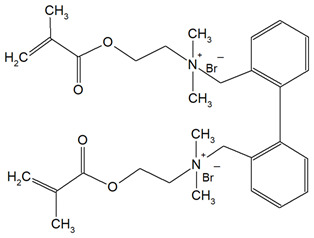		[65,66,67]
IMQ-16 (n = 15)	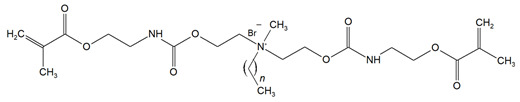	Degree of conversion (DC); Water sorption (WS); Water solubility (SL); Flexural strength (FS); Flexural modulus (E); Direct contact test (DCT).	[68]
QABGMA	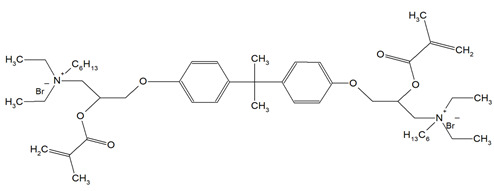	Degree of conversion (DC); Water sorption (WS); Water solubility (SL); Antimicrobial activity; Cytotoxicity.	[69]
MAE-HB	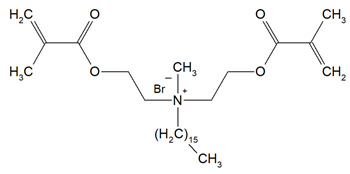	Antibacterial activity.	[70]

**Table 2:** Mechanical and physicochemical properties of QADMs incorporated into copolymers and composites.

Copolymer	Composite	Mechanical Properties				Physicochemical Properties							Ref.
		Hardness, [MPa]	E, [MPa]	FS, [MPa]	CS, [MPa]	WS, [μg/mm^3^]	SL, [μg/mm^3^]	DC, [%]	S_e_, [%]	S_T_, [%]	T_g_, [°C]	WCA, [°]	
40 wt.% (QAn + IPDI) + 40 wt.% Bis-GMA + 20 wt.% TEGDMA		-	-	-	-	QA8 = 14.00 ± 1.61, QA10 = 13.18 ± 1.02	QA8 = 2.70 ± 0.29, QA10 = 2.62 ± 0.26	-	-	-	-	-	[55]
40 wt.% (QAn + CHMDI) + 40 wt.% Bis-GMA + 20 wt.% TEGDMA		-	-	-	-	QA8 = 13.75 ± 1.32, QA10 = 11.79 ± 0.14	QA8 = 2.94 ± 0.21, QA10 = 3.08 ± 0.20	-	-	-	-	-	
40 wt.% (QAn + MDI) + 40 wt.% Bis-GMA + 20 wt.% TEGDMA		-	-	-	-	QA8 = 11.67 ± 0.32, QA10 = 11.63 ± 0.47	QA8 = 7.68 ± 0.48, QA10 = 6.61 ± 0.53	-	-	-	-	-	
20 wt.% (QAn + TMXDI) + 20 wt.% UDMA + 40 wt.% Bis-GMA + 20 wt.% TEGDMA		QA10 = 234.8 ± 8.7, QA12 = 194.6 ± 26.5	QA10 = 3244.9 ± 114.1, QA12 = 3020.6 ± 198.9	QA10 = 100.6 ± 11.7, QA12 = 90.3 ± 6.1	-	QA10 = 10.43 ± 0.42, QA12 = 10.35 ± 0.23	QA10 = 2.18 ± 0.06, QA12 = 2.46 ± 0.25	QA10 = 57.0 ± 5.0, QA12 = 69.0 ± 6.0	QA10 = 7.04 ± 0.83, QA12 = 7.72 ± 1.17	-	QA10 = 53.46 ± 3.27, QA12 = 60.30 ± 4.34	QA10 = 87.03 ± 3.07, QA12 = 91.30 ± 4.82	[50]
40 wt.% (QAUDMA-m) + 40 wt.% Bis-GMA + 20 wt.% TEGDMA		m:8 = 153.91 ± 5.56, m:10 = 128.21 ± 8.08, m:12 = 126.96 ± 3.98, m:14 = 113.63 ± 5.25, m:16 = 98.13 ± 3.04, m:18 = 83.84 ± 2.85	m:8 = 3716.68 ± 90.76, m:10 = 3393.39 ± 83.10, m:12 = 2958.97 ± 177.27, m:14 = 2636.08 ± 190.78, m:16 = 2181.70 ± 164.70, m:18 = 1986.74 ± 58.03	m:8 = 74.47 ± 4.67, m:10 = 65.25 ± 3.63, m:12 = 60.01 ± 3.75, m:14 = 55.58 ± 3.02, m:16 = 54.75 ± 3.48, m:18 = 50.81 ± 4.96	-	m:8 = 68.27, m:10 = 48.42, m:12 = 35.54, m:14 = 34.43, m:16 = 32.67, m:18 = 25.94	m:8 = 5.15, m:10 = 5.18, m:12 = 5.22, m:14 = 5.58, m:16 = 5.42, m:18 = 5.54	m:8 = 59.0 ± 2.0, m:10 = 60.0 ± 2.0, m:12 = 61.0 ± 2.0, m:14 = 63.0 ± 2.0, m:16 = 66.0 ± 1.0, m:18 = 68.0 ± 2.0	m:8 = 5.08 ± 0.40, m:10 = 5.48 ± 0.37, m:12 = 6.07 ± 0.49, m:14 = 6.14 ± 0.41, m:16 = 6.24 ± 0.54, m:18 = 6.40 ± 0.48	m:8= 9.81, m:10= 9.60, m:12= 9.38, m:14= 9.18, m:16= 9.04, m:18 = 8.90	m:8 = 42.21 ± 1.34, m:10 = 45.81 ± 1.17, m:12 = 46.63 ± 1.12, m:14 = 47.83 ± 1.08, m:16 = 50.41 ± 1.87, m:18 = 50.81 ± 0.95	m:8 = 81.41 ± 1.57, m:10 = 84.68 ± 2.36, m:12 = 86.32 ± 1.63, m:14 = 85.52 ± 1.40, m:16 = 91.05 ± 1.24, m:18 = 99.53 ± 1.62	[56,57]
60 wt.% (QAUDMA-m) + 40 wt.% TEGDMA		m:8 = 51.41 ± 4.32, m:10 = 51.17 ± 6.93, m:12 = 50.87 ± 4.08, m:14 = 41.60 ± 3.63, m:16 = 41.21 ± 2.27, m:18 = 42.17 ± 1.08	m:8 = 679.0 ± 36.2, m:10 = 851.6 ± 47.4, m:12 = 848.7 ± 24.7, m:14 = 772.3 ± 31.1, m:16 = 753.5 ± 31.8, m:18 = 459.4 ± 34.4	m:8 = =21.59 ± 0.66, m:10 = 37.37 ± 2.27, m:12 = 34.46 ± 2.18, m:14 = 28.38 ± 1.38, m:16 = 20.13 ± 1.62, m:18 = 21.75 ± 1.90	-	m:8 = 148.31, m:10 = 138.42, m:12 = 130.67, m:14 = 124.89, m:16 = 121.21, m:18 = 116.08	m:8 = 52.39, m:10 = 32.23, m:12 = 24.21, m:14 = 19.08, m:16 = 15.41, m:18 = 12.67	m:8 = 84.2 ± 1.2, m:10 = 84.0 ± 0.9, m:12 = 86.0 ± 1.2, m:14 = 88.7 ± 1.4, m:16 = 87.1 ± 1.1, m:18 = 87.1 ± 0.9	m:8 = 6.6 ± 0.3, m:10 = 6.4 ± 0.3, m:12 = 6.5 ± 0.6, m:14 = 6.9 ± 0.6, m:16 = 6.5 ± 1.0, m:18 = 6.5 ± 0.9	m:8 = 10.4, m:10 = 10.0, m:12 = 9.7, m:14 = 9.4, m:16 = 9.2, m:18 = 9.0	m:8 = 60.52 ± 0.79, m:10 = 60.33 ± 1.37, m:12 = 63.18 ± 1.43, m:14 = 64.07 ± 1.15, m:16 = 65.03 ± 0.73, m:18 = 66.32 ± 1.23	m:8 = 82.1 ± 2.7, m:10 = 82.6 ± 2.4, m:12 = 84.4 ± 1.7, m:14 = 86.1 ± 1.7, m:16 = 94.7 ± 2.3, m:18 = 98.7 ± 2.1	[49,58]
10 wt.% Bis-QADM-Bet + 40 wt.% Bis-GMA + 50 wt.% TEGDMA		-	EBet ~2.2 × 10^3^ *, BBet ~2.5 × 10^3^ *, HBet ~2.3 × 10^3^ *	EBet ~70 *, BBet ~110.0 *, HBet ~90.0 *	EBet ~290 *, BBet ~310 *, HBet ~280 *	-	-	EBet = 43.4, BBet = 45.6, HBet = 47.52	-	-	-	EBet = 68.0, BBet = 93.0, HBet = 98.0	[59]
QANMA + Bis-GMA/TEGDMA (50/50 wt.)		-	5 wt.% = (1.90 ± 0.14) × 10^3^–(2.64 ± 0.09) × 10^3^, 10 wt.% = (1.73 ± 0.20) × 10 ^3^–(2.43 ± 0.12) × 10^3^, 20 wt.% = (1.18 ± 0.10) × 10^3^–(2.14 ± 0.14) × 10^3^	5 wt.% = 84 ± 7–119 ± 4, 10 wt.% = 77 ± 9–108 ± 4, 20 wt.% = 47 ± 9–92 ± 6	-	5 wt.% = 6.9 ± 0.6 ^#^, 10 wt.% = 7.6 ± 0.7 ^#^, 20 wt.% = 47 ± 9–92 ± 6 ^#^	5 wt.% = 2.5 ± 0.4 ^#^, 10 wt.% = 3.2 ± 0.7 ^#^, 20 wt.% = 4.1 ± 0.7 ^#^	5 wt.% = 65.5 ± 3.3, 10 wt.% = 66.4 ± 3.1, 20 wt.% = 65.0 ± 1.8	5 wt.% = 9.2 ± 0.4, 10 wt.% = 8.9 ± 0.5, 20 wt.% = 7.1 ± 1.2	-	-	-	[61]
1 wt.% DMBH + 99 wt.% Tetric N-Bond		-	-	-	-	-	-	~78.0 *	-	-	-	-	[62]
1 wt.% DMBB + 99 wt.% Tetric N-Bond		-	-	-	-	-	-	~79.0 *	-	-	-	-	
	(30 wt.% UDMQA-12 + 19.3 wt.% Bis-GMA + 9.3 wt.% TEGDMA) + silanated glass (30:70)	-	~7.8 × 10^3^ *	~135.0 *	-	-	-	-	-	-	-	-	[64]
IDMA-1 + 50.91 wt.% UDMA +18.18 wt.% PEG-U + 30.91 wt.% EHMA (UPE resin)		-	-	10 wt.% ~180.0 *, 20 wt.% ~180.0 *	-	-	-	10 wt.% ~92.0 *, 20 wt.% ~92.0 *	-	-	-	10 wt.% ~65.0 *, 20 wt.% ~63.0 *	[65]
	60 wt.% UPE-IDMA-1 + 40 wt.% ACP filler	-	-	~60.0 *	-	-	-	~87.0 *	-	-	-	-	
IDMA-2 + 50.91 wt.% UDMA +18.18 wt.% PEG-U + 30.91 wt.% EHMA (UPE resin)		-	-	10 wt.% ~182.0 *, 20 wt.% ~182.0 *	-	-	-	10 wt.% ~93.0 *, 20 wt.% ~93.0 *	-	-	-	10 wt.% ~64 *, 20 wt.% ~66 *	
	60 wt.% UPE-IDMA-2 + 40 wt.% ACP filler	-	-	~80 *	-	-	-	~94.0 *	-	-	-	-	
IMQ-16 + UDMA + SR833s		-	5 wt.% = 2.36 ± 0.08–2.77 ± 0.07, 8 wt.% = 2.44 ± 0.08–2.75 ± 0.10, 11 wt.% = 2.45 ± 0.06–2.75 ± 0.06, 14 wt.% = 2.32 ± 0.09–2.64 ± 0.09, 17 wt.% = 2.31 ± 0.06–2.65 ± 0.06, 20 wt.% = 2.16 ± 0.09–2.49 ± 0.08	5 wt.% = 104.5 ± 5.1–114.1 ± 15.5, 8 wt.% = 97.6 ± 12.5–120.7 ± 15.5, 11 wt.% = 101.1 ± 14.5–110.1 ± 12.9, 14 wt.% = 91.9 ± 12.8–110.3 ± 15.3, 17 wt.% = 94.0 ± 13.5–111.8 ± 15.2, 20 wt.% = 79.4 ± 5.6–97.4 ± 12.1	-	5 wt.% = 2.30 ± 0.02 ^#^, 8 wt.% = 2.58 ± 0.02 ^#^, 11 wt.% = 2.83 ± 0.01 ^#^, 14 wt.% = 3.06 ± 0.01 ^#^, 17 wt.% = 3.28 ± 0.01 ^#^, 20 wt.% = 3.80 ± 0.02 ^#^	5 wt.% = 0.36 ± 0.02 ^#^, 8 wt.% = 0.42 ± 0.03 ^#^, 11 wt.% = 0.43 ± 0.02 ^#^, 14 wt.% = 0.53 ± 0.02 ^#^, 17 wt.% = 0.58 ± 0.05 ^#^, 20 wt.% = 0.98 ± 0.03 ^#^	5 wt.% = 69.1 ± 0.54, 8 wt.% = 69.0 ± 0.44, 11 wt.% = 68.4 ± 0.57, 14 wt.% = 68.1 ± 0.71, 17 wt.% = 68.6 ± 0.79, 20 wt.% = 72.6 ± 1.12	-	-	-	-	[68]
QABGMA + Bis-GMA/TEGDMA (50/50 wt.)		-	-	-	-	5 wt.% = 2.06–6.98 ^#^, 10 wt.% = 2.54–7.86 ^#^, 15 wt.% = 3.1–9.68 ^#^	5 wt.% = 0.49–3.25 ^#^, 10 wt.% = 0.55–3.30 ^#^, 15 wt.% = 0.56–3.38 ^#^	5 wt.% ~69.0 *, 10 wt.% ~68.0 *, 15 wt.% ~67.0 *	-	-	-	-	[69]

* Data extracted from figures in referenced sources. ^#^ Values reported from referenced sources expressed as percentages.

## Data Availability

No new data were created or analyzed in this study. Data sharing is not applicable to this article.

## Supplementary Materials

The following supporting information can be downloaded at: https://www.mdpi.com/article/10.3390/ma18214844/s1, Table S1: Keyword occurrence of QADMs and their total link strength; Table S2: Summary of antimicrobial activity of QADMs against different microbial strains. References [50,51,56,59,60,61,62,63,64,66,69,70] are cited in Supplementary Materials.

## References

[1] Karadag M., Dolekcekic E., Erdem M., Özcan M. (2024). Effect of Stearyl Methacrylate Comonomer on the Mechanical and Physical Properties of Dimethacrylate-Based Dental Resins. Materials.

[2] Liu Z., Jia T., Yang Y., Yue X., Liu Y., Zhang X., Chen Y., Ma S., Valenzuela C., Wang L. (2024). Near–Infrared Light–Cured Dental Restoration Materials with Upconversion Nanoparticles. Chem. Eng. J..

[3] Cho K., Rajan G., Farrar P., Prentice L., Prusty B.G. (2022). Dental Resin Composites: A Review on Materials to Product Realizations. Compos. Part B Eng..

[4] Dursun E., Fron-Chabouis H., Attal J.-P., Raskin A. (2016). Bisphenol A Release: Survey of the Composition of Dental Composite Resins. Open Dent. J..

[5] Pawłowska E., Loba K., Błasiak J., Szczepanska J. (2009). Properties and Risk of the Use of Bisphenol A−Glycidyl Methacrylate and Urethane Dimethacrylate–Basic Monomers of Dental Restorative Materials. Dent. Med. Probl..

[6] Asmussen E., Peutzfeldt A. (1998). Influence of UEDMA BisGMA and TEGDMA on Selected Mechanical Properties of Experimental Resin Composites. Dent. Mater. Off. Publ. Acad. Dent. Mater..

[7] Peutzfeldt A. (1997). Resin Composites in Dentistry: The Monomer Systems. Eur. J. Oral Sci..

[8] Gonçalves F., Pfeifer C.C.S., Stansbury J.W., Newman S.M., Braga R.R. (2010). Influence of Matrix Composition on Polymerization Stress Development of Experimental Composites. Dent. Mater. Off. Publ. Acad. Dent. Mater..

[9] Dulik D., Bernier R., Brauer G.M. (1981). Effect of Diluent Monomer on the Physical Properties of Bis-GMA-Based Composites. J. Dent. Res..

[10] Aminoroaya A., Neisiany R.E., Khorasani S.N., Panahi P., Das O., Madry H., Cucchiarini M., Ramakrishna S. (2021). A Review of Dental Composites: Challenges, Chemistry Aspects, Filler Influences, and Future Insights. Compos. Part B Eng..

[11] Hamama H. (2019). Recent Advances in Posterior Resin Composite Restorations. Appl. Nanocomposite Mater. Dent..

[12] Schricker S.R., Eliades T., Brantley W.A. (2017). 9-Composite Resin Polymerization and Relevant Parameters. Orthodontic Applications of Biomaterials.

[13] Abdulsamee N. (2020). Shrinkage of Dental Composite Resin: Contemporary Understanding Its Enigmas and How to Solve? A Review. EC Dent. Sci..

[14] Padovani G.C., Fùcio S.B.P., Ambrosano G.M.B., Correr-Sobrinho L., Puppin-Rontani R.M. (2015). In Situ Bacterial Accumulation on Dental Restorative Materials. CLSM/COMSTAT Analysis. Am. J. Dent..

[15] Reis A.F., Giannini M., Lovadino J.R., dos Santos Dias C.T. (2002). The Effect of Six Polishing Systems on the Surface Roughness of Two Packable Resin-Based Composites. Am. J. Dent..

[16] Chisini L.A., Collares K., Cademartori M.G., de Oliveira L.J.C., Conde M.C.M., Demarco F.F., Corrêa M.B. (2018). Restorations in Primary Teeth: A Systematic Review on Survival and Reasons for Failures. Int. J. Paediatr. Dent..

[17] Bernardo M., Luis H., Martin M.D., Leroux B.G., Rue T., Leitão J., DeRouen T.A. (2007). Survival and Reasons for Failure of Amalgam versus Composite Posterior Restorations Placed in a Randomized Clinical Trial. J. Am. Dent. Assoc. 1939.

[18] Alhareky M., Tavares M. (2016). Amalgam vs Composite Restoration, Survival, and Secondary Caries. J. Evid.-Based Dent. Pract..

[19] Pirmoradian M., Hooshmand T., Asiri A.M., Inamuddin Mohammad A. (2019). 15-Remineralization and Antibacterial Capabilities of Resin-Based Dental Nanocomposites. Applications of Nanocomposite Materials in Dentistry.

[20] Stencel R., Kasperski J., Pakieła W., Mertas A., Bobela E., Barszczewska-Rybarek I., Chladek G. (2018). Properties of Experimental Dental Composites Containing Antibacterial Silver-Releasing Filler. Materials.

[21] Mallineni S.K., Sakhamuri S., Kotha S.L., AlAsmari A.R.G.M., AlJefri G.H., Almotawah F.N., Mallineni S., Sajja R. (2023). Silver Nanoparticles in Dental Applications: A Descriptive Review. Bioengineering.

[22] Mirhashemi A.H., Bahador A., Kassaee M.Z., Daryakenari G., Ahmad-Akhoundi M.S., Sodagar A. (2013). Antimicrobial Effect of Nano-Zinc Oxide and Nano-Chitosan Particles in Dental Composite Used in Orthodontics. J. Med. Bacteriol..

[23] Song W., Ge S. (2019). Application of Antimicrobial Nanoparticles in Dentistry. Molecules.

[24] Bapat R.A., Joshi C.P., Bapat P., Chaubal T.V., Pandurangappa R., Jnanendrappa N., Gorain B., Khurana S., Kesharwani P. (2019). The Use of Nanoparticles as Biomaterials in Dentistry. Drug Discov. Today.

[25] Fathi M., Hosseinali Z., Molaei T., Hekmatfar S. (2024). The Effect of Silver and Calcium Fluoride Nanoparticles on Antibacterial Activity of Composite Resin against Streptococcus Mutans: An in Vitro Study. Dent. Res. J..

[26] Chladek G., Barszczewska-Rybarek I., Chrószcz-Porębska M., Mertas A. (2023). The Effect of Quaternary Ammonium Polyethylenimine Nanoparticles on Bacterial Adherence, Cytotoxicity, and Physical and Mechanical Properties of Experimental Dental Composites. Sci. Rep..

[27] Stein K., Farmer J., Singhal S., Marra F., Sutherland S., Quiñonez C. (2018). The Use and Misuse of Antibiotics in Dentistry: A Scoping Review. J. Am. Dent. Assoc. 1939.

[28] Vardanyan R., Hruby V. (2016). Antibiotics. Synthesis of Best-Seller Drugs.

[29] Malekhoseini Z., Rezvani M.B., Niakan M., Atai M., Bassir M.M., Alizade H.S., Siabani S. (2021). Effect of Zinc Oxide Nanoparticles on Physical and Antimicrobial Properties of Resin-Modified Glass Ionomer Cement. Dent. Res. J..

[30] Yu Y., Xia F., Liu R., Yan Y., Yin L. (2024). Effect of Calcium Supplementation and TMEM16A Inhibition on Endoplasmic Reticulum Stress Induced by Dental Fluorosis in Mice. Discov. Med..

[31] Ma X., Lin X., Zhong T., Xie F. (2019). Evaluation of the Efficacy of Casein Phosphopeptide-Amorphous Calcium Phosphate on Remineralization of White Spot Lesions in Vitro and Clinical Research: A Systematic Review and Meta-Analysis. BMC Oral Health.

[32] Kianfar E., Mahmoud Z. (2024). Effect and Investigating of Graphene Nanoparticles on Mechanical, Physical Properties of Polylactic Acid Polymer. Case Stud. Chem. Environ. Eng..

[33] Schwartz A.B., Larson E.L. (2007). Antibiotic Prophylaxis and Postoperative Complications after Tooth Extraction and Implant Placement: A Review of the Literature. J. Dent..

[34] Aslam B., Wang W., Arshad M.I., Khurshid M., Muzammil S., Rasool M.H., Nisar M.A., Alvi R.F., Aslam M.A., Qamar M.U. (2018). Antibiotic Resistance: A Rundown of a Global Crisis. Infect. Drug Resist..

[35] Ventola C.L. (2015). The Antibiotic Resistance Crisis: Part 1: Causes and Threats. Pharm. Ther..

[36] Carmona-Ribeiro A.M., Araújo P.M. (2021). Antimicrobial Polymer−Based Assemblies: A Review. Int. J. Mol. Sci..

[37] Pandit G., Chowdhury N., Abdul Mohid S., Bidkar A.P., Bhunia A., Chatterjee S. (2021). Effect of Secondary Structure and Side Chain Length of Hydrophobic Amino Acid Residues on the Antimicrobial Activity and Toxicity of 14-Residue-Long de Novo AMPs. ChemMedChem.

[38] Cao J., Ma Q., Shi J., Wang X., Ye D., Liang J., Zou J. (2025). Cariogenic Microbiota and Emerging Antibacterial Materials to Combat Dental Caries: A Literature Review. Pathogens.

[39] Arnold W.A., Blum A., Branyan J., Bruton T.A., Carignan C.C., Cortopassi G., Datta S., DeWitt J., Doherty A.-C., Halden R.U. (2023). Quaternary Ammonium Compounds: A Chemical Class of Emerging Concern. Environ. Sci. Technol..

[40] Quaternary Ammonium Compounds in Cleaning Products: Health & Safety Information for Health Professionals. https://www.mountsinai.org/files/MSHealth/Assets/HS/Patient-Care/Service-Areas/Occupational-Medicine/QACsInfoforPhysicians_18.pdf.

[41] An J., Gao J., Zhao J., Cui Y., Zeng L., Xu H., Wang Q. (2024). Quaternary Ammonium Compounds Inhibited Phosphorus Removal Performance and Aggravated the Spread of Resistance Genes in Enhanced Biological Phosphorus Removal Systems. Chem. Eng. J..

[42] US EPA O. About List N: Disinfectants for Coronavirus (COVID-19). https://www.epa.gov/coronavirus-and-disinfectants/about-list-n-disinfectants-coronavirus-covid-19.

[43] Jabar S., Kumar S., Neena A. (2020). Effectiveness of Various Disinfectant Protocols on Orthodontic Pliers—An In-Vitro Study. Int. J. Med. Dent. Sci..

[44] Gilbert P., Moore L.E. (2005). Cationic Antiseptics: Diversity of Action under a Common Epithet. J. Appl. Microbiol..

[45] Makvandi P., Jamaledin R., Jabbari M., Nikfarjam N., Borzacchiello A. (2018). Antibacterial Quaternary Ammonium Compounds in Dental Materials: A Systematic Review. Dent. Mater. Off. Publ. Acad. Dent. Mater..

[46] Kihara T., Ikawa T., Shigeta Y., Shigemoto S., Ihara K., Sasaki K., Hirai K., Ogawa T. (2022). Considerations for the Selection of Interim Restoration Materials Using Wear Test Results. J. Prosthodont. Res..

[47] Nikolaidis A.K., Koulaouzidou E.A., Gogos C., Achilias D.S. (2021). Synthesis of Novel Dental Nanocomposite Resins by Incorporating Polymerizable, Quaternary Ammonium Silane-Modified Silica Nanoparticles. Polymers.

[48] Chladek G. (2023). Composite and Polymeric Materials for Dentistry: Enhancing Antimicrobial and Mechanical Properties. Materials.

[49] Chrószcz M.W., Barszczewska-Rybarek I.M., Kazek-Kęsik A. (2022). Novel Antibacterial Copolymers Based on Quaternary Ammonium Urethane-Dimethacrylate Analogues and Triethylene Glycol Dimethacrylate. Int. J. Mol. Sci..

[50] Drejka P., Chrószcz-Porębska M., Kazek-Kęsik A., Chladek G., Barszczewska-Rybarek I. (2024). Chemical Modification of Dental Dimethacrylate Copolymer with Tetramethylxylylene Diisocyanate-Based Quaternary Ammonium Urethane-Dimethacrylates—Physicochemical, Mechanical, and Antibacterial Properties. Materials.

[51] Chrószcz-Porębska M.W., Barszczewska-Rybarek I.M., Kazek-Kęsik A., Ślęzak-Prochazka I. (2023). Cytotoxicity and Microbiological Properties of Copolymers Comprising Quaternary Ammonium Urethane-Dimethacrylates with Bisphenol A Glycerolate Dimethacrylate and Triethylene Glycol Dimethacrylate. Materials.

[52] Pantea M., Totan A.R., Imre M., Petre A.E., Țâncu A.M.C., Tudos C., Farcașiu A.T., Butucescu M., Spînu T.C. (2021). Biochemical Interaction between Materials Used for Interim Prosthetic Restorations and Saliva. Materials.

[53] EU Moves to Ban Mercury Uses in Dental Amalgams by 2025. https://ecomundo.eu/en/blog/eu-ban-mercury-dental-amalgams-2025.

[54] van Eck N.J., Waltman L. (2019). VOSviewer Manual.

[55] Drejka P., Kula P., Barszczewska-Rybarek I. (2025). Novel Quaternary Ammonium Urethane-Dimethacrylates for Copolymers with Low Water Sorption and Solubility. Molecules.

[56] Chrószcz-Porębska M., Kazek-Kęsik A., Chladek G., Barszczewska-Rybarek I. (2023). Novel Mechanically Strong and Antibacterial Dimethacrylate Copolymers Based on Quaternary Ammonium Urethane-Dimethacrylate Analogues. Dent. Mater..

[57] Chrószcz-Porębska M.W., Barszczewska-Rybarek I.M., Chladek G. (2023). Physicochemical Properties of Novel Copolymers of Quaternary Ammonium UDMA Analogues, Bis-GMA, and TEGDMA. Int. J. Mol. Sci..

[58] Chrószcz-Porębska M.W., Barszczewska-Rybarek I.M., Chladek G. (2022). Characterization of the Mechanical Properties, Water Sorption, and Solubility of Antibacterial Copolymers of Quaternary Ammonium Urethane-Dimethacrylates and Triethylene Glycol Dimethacrylate. Materials.

[59] Zhang L., Ma Z., Wang R., Zuo W., Zhu M. (2023). Bis-Quaternary Ammonium Betulin-Based Dimethacrylate: Synthesis, Characterization, and Application in Dental Restorative Resins. Mater. Adv..

[60] Fanfoni L., Marsich E., Turco G., Breschi L., Cadenaro M. (2021). Development of Di-Methacrylate Quaternary Ammonium Monomers with Antibacterial Activity. Acta Biomater..

[61] Li S., Yu X., Liu F., Deng F., He J. (2020). Synthesis of Antibacterial Dimethacrylate Derived from Niacin and Its Application in Preparing Antibacterial Dental Resin System. J. Mech. Behav. Biomed. Mater..

[62] Manouchehri F., Sadeghi B., Najafi F., Mosslemin M.H., Niakan M. (2019). Synthesis and Characterization of Novel Polymerizable Bis-Quaternary Ammonium Dimethacrylate Monomers with Antibacterial Activity as an Efficient Adhesive System for Dental Restoration. Polym. Bull..

[63] Wang Y., Costin S., Zhang J., Liao S., Wen Z.T., Lallier T., Yu Q., Xu X. (2018). Synthesis, Antibacterial Activity, and Biocompatibility of New Antibacterial Dental Monomers. Am. J. Dent..

[64] Huang Q., Huang S., Liang X., Qin W., Liu F., Lin Z., He J. (2018). The Antibacterial, Cytotoxic, and Flexural Properties of a Composite Resin Containing a Quaternary Ammonium Monomer. J. Prosthet. Dent..

[65] Bienek D.R., Frukhtbeyn S.A., Giuseppetti A.A., Okeke U.C., Skrtic D. (2018). Antimicrobial Monomers for Polymeric Dental Restoratives: Cytotoxicity and Physicochemical Properties. J. Funct. Biomater..

[66] Cheng L., Zhang K., Zhou C.-C., Weir M.D., Zhou X.-D., Xu H.H.K. (2016). One-Year Water-Ageing of Calcium Phosphate Composite Containing Nano-Silver and Quaternary Ammonium to Inhibit Biofilms. Int. J. Oral Sci..

[67] Antonucci J.M., Zeiger D.N., Tang K., Lin-Gibson S., Fowler B.O., Lin N.J. (2012). Synthesis and Characterization of Dimethacrylates Containing Quaternary Ammonium Functionalities for Dental Applications. Dent. Mater..

[68] Huang Q., He J., Lin Z., Liu F., Lassila L.V.J., Vallittu P.K. (2016). Physical and Chemical Properties of an Antimicrobial Bis-GMA Free Dental Resin with Quaternary Ammonium Dimethacrylate Monomer. J. Mech. Behav. Biomed. Mater..

[69] Makvandi P., Ghaemy M., Mohseni M. (2016). Synthesis and Characterization of Photo-Curable Bis-Quaternary Ammonium Dimethacrylate with Antimicrobial Activity for Dental Restoration Materials. Eur. Polym. J..

[70] Huang L., Yu F., Sun X., Dong Y., Lin P., Yu H., Xiao Y., Chai Z., Xing X., Chen J. (2016). Antibacterial Activity of a Modified Unfilled Resin Containing a Novel Polymerizable Quaternary Ammonium Salt MAE-HB. Sci. Rep..

[71] Sommers K.J., Bentley B.S., Carden R.G., Post S.J., Allen R.A., Kontos R.C., Black J.W., Wuest W.M., Minbiole K.P.C. (2021). Metallocene QACs: The Incorporation of Ferrocene Moieties into monoQAC and bisQAC Structures. ChemMedChem.

[72] Macias-Paz I.U., Pérez-Hernández S., Tavera-Tapia A., Luna-Arias J.P., Guerra-Cárdenas J.E., Reyna-Beltrán E. (2023). Candida albicans the main opportunistic pathogenic fungus in humans. Rev. Argent. Microbiol..

[73] Patel M. (2022). Oral Cavity and Candida Albicans: Colonisation to the Development of Infection. Pathogens.

[74] (2009). Biological Evaluation of Medical Devices-Part 5: Tests for In Vitro Cytotoxicity.

[75] (2019). Dentistry—Polymer-Based Restorative Materials.

[76] Barszczewska-Rybarek I.M. (2019). A Guide through the Dental Dimethacrylate Polymer Network Structural Characterization and Interpretation of Physico-Mechanical Properties. Materials.

[77] Zhao H., Cai S., Hua R., Li C., Xia C., Cui B., Shao H., Bu N., Yuan Y. (2025). In Situ Polymerization of Long Alkyl Chain Functional Groups Enhances the Oil–Water Separation Performance of Porous Organic Polymers. Molecules.

[78] Sun Q., Zhang L., Bai R., Zhuang Z., Zhang Y., Yu T., Peng L., Xin T., Chen S., Han B. (2021). Recent Progress in Antimicrobial Strategies for Resin-Based Restoratives. Polymers.

[79] Wang W., Wu F., Zhang G., Zhu S., Ban J., Wang L. (2019). Preparation of a Highly Crosslinked Biosafe Dental Nanocomposite Resin with a Tetrafunctional Methacrylate Quaternary Ammonium Salt Monomer. RSC Adv..

[80] Ramić A., Skočibušić M., Odžak R., Čipak Gašparović A., Milković L., Mikelić A., Sović K., Primožič I., Hrenar T. (2021). Antimicrobial Activity of Quasi-Enantiomeric Cinchona Alkaloid Derivatives and Prediction Model Developed by Machine Learning. Antibiotics.

