# Supplementary material for: Quaternary Ammonium Dimethacrylates as an Additive in Dental Composite Resins: A Review of Their Antimicrobial, Mechanical, and Physicochemical Properties
Source: Materials (Basel). 2025 Oct 23;18(21):4844. doi: 10.3390/ma18214844 (PMC12610268; doi:10.3390/ma18214844)
Supplement: Supplementary file 1 [file materials-18-04844-s001.zip › materials-3911159-supplementary.pdf]

Supplementary Information.

Table S1. Keyword occurrence of QADMs and their total link strength.

Keyword	Occurrences	Total link strength
Antibacterial activity	15	86
Cytotoxicity	12	58
Dental resin	11	60
Quaternary ammonium	11	42
Monomers	9	59
Antibacterial	9	27
Conversion	8	44
Adhesive	7	49
Composites	7	33
Physicochemical properties	6	38
Caries	6	34
Mechanical-properties	6	33
Restorative materials	6	29
Nanoparticles	6	20
Mechanical properties	5	35
Chemical-properties	5	34
Quaternary ammonium compounds	5	28
Water sorption	5	28
Dimethacrylate	5	24
Antimicrobial activity	5	23
Resin	5	20
Silver nanoparticles	5	18
Urethane-dimethacrylates	4	24
Streptococcus-mutans	4	22
Amalgam	4	20
Composite	4	18
Polymerization	4	17
Biofilm	4	16
Polymers	4	5

Table S2. Summary of antimicrobial activity of QADMs against different microbial strains.

Study	Method of Antimicrobial Validation	Strains Tested	Antimicrobial activity
Drejka et al. [1]	• MIC/MBC tests	• *S. aureus - ATCC 25923*<br>• *E. coli - ATCC 25922*	1. 20(QA10+TMXDI)p exhibited the strongest activity against both strains (MIC = 6.25 mg/mL, MBC = 12.5 mg/mL).

			2. 20(QA12+TMXDI)p was less effective against S. aureus (MIC/MBC > 50 mg/mL), but moderately effective against E. coli (MIC = 12.5 mg/mL, MBC = 24 mg/mL)<br>3. 40(UDMA)p had the weakest antimicrobial activity against both strains (MIC/MBC > 50 mg/mL)<br>4. Extending the N-alkyl chain in QAUDMA (from C10 to C12) reduced antibacterial potency.
Chrószcz-Porębska et al. [2]	• Bacterial adhesion test<br>• IZD test	• *S. aureus - ATCC 25923*<br>• *E. coli - ATCC 25922*	1. No bacterial growth observed on BG:QA8:TEG and BG:QA10:TEG surfaces; BG:QA12:TEG fully inhibited *E. coli*.<br>2. On other BG:QAm:TEG samples, bacterial adhesion increased in higher alkyl chain length: *S. aureus* (3.16 – 6.47 logCFU/mL) and *E.coli* (4.40 – 4.99 logCFU/mL)<br>3. IZD ranged between 10 – 23 mm for *S. aureus* and 12 -21 mm for *E.coli* and decreased with increasing alkyl chain length.
Chrószcz-Porębska et al. [3]	• Fungal adhesion/ growth inhibition tests<br>• IZD test	• *C. albicans - ATCC 2091*<br>• *S. aureus - ATCC 25923*<br>• *E. coli - ATCC 25922*	1. *C. albicans* on BG:QAm:TEG surfaces ranged from 3.20 – 4.15 logCFU/mL.<br>2. Inhibition zones (13 – 7 mm) observed only for alkyl chains below C16, and decreasing with increasing chain length.<br>3. Agar plate assays (25 mg/mL suspensions) showed complete inhibition of all strains.
Zhang et al. [4]	• Live/Dead Biofilm staining<br>• Protein leakage and ion-release analysis	• *S. mutans*	1. Control resin (5B5T) showed dense S. mutans colonies; experimental resins (1EBet4B5T, 1BBet4B5T, 1HBet4B5T) had no

	• Gene expression analysis (RT-qPCR)		viable bacteria (>99% antibacterial efficiency)<br>2. Alkyl chain length of C8 provided optimal charge density for contact killing; C12 reduced likely due to chain curling.<br>3. TEM and leakage assays displayed increased protein (0.6 – 0.7 mg/mL) and ion release, particularly with 1BBet4B5T (highest K+, FE3+, Mg2+), indicating severe cell damage.<br>4. 1BBet4B5T reduced *gtfB* and *gtfC* expression (biofilm-related genes).
Fanfoni et al. [5]	• MIC/MBC tests<br>• Biofilm inhibition test	• *S. mutans - ATCC 25175*<br>• *E. coli - ATCC 25922*<br>• *S. aureus - ATCC 25923*<br>• *S. sanguinis - ATCC 10556*<br>• *S. mitis - ATCC 49456*	1. Nine new QADMs synthesized and compared with commercial MDPB and IDMA-2.<br>2. DDPyMMA exhibited strongest activity against *S.mutans* (MIC/MBC = 2.5 μg/mL)<br>3. DDMAPMA showed highest activity against remaining strains.<br>4. All QADMs significantly reduced biofilm formation, even at MIC concentrations.
Li et al. [6]	• Live/Dead staining	• *S. mutans - ATCC 25175*	1. 10% and 20% QANMA resin incorporation reduced *S. mutans* CFU; 5% showed no significant antibacterial effect.<br>2. Live/dead assay confirmed fewer live bacteria with increasing QANMA content.<br>3. 20% QANMA exhibited the strongest bactericidal effect with the deadest cells.
Manouchehri et al. [7]	• MIC test<br>• IZD test	• *Streptococcus mutans*	1. Addition of DMBH and DMBB lowered MIC to 3.12 μg/mL and 6.25 μg/mL respective, as compared to the control adhesive (2.4 x 10^3 μg/mL).<br>2. Control + DMBH exhibited the largest inhibition zones (9 mm),

			followed by control + DMBB (7 mm) and the control (2 mm)
Wang et al. [8]	• Growth inhibition test	• *S. mutans - UA159*<br>• *L. casei - ATCC 4646*<br>• *S. aureus - ATCC 25923*<br>• *P. aeruginosa - ATCC 27853*	1. DPHB and DPNB showed strong antibacterial activity against *S.mutans*, *S. aureus*, and *L. casei* at concentrations ≥ 10^{-5} M, and against *P. aeruginosa* at concentrations ≥ 10^{-4} M.<br>2. DPDB was effective against *L. casei* at 10^{-4} concentrations but limited against other strains.
Huang et al. [9]	• Direct contact test	• *S. mutans*	1. Filtek Z250 composite and Fuji VII GIC served as negative and positive controls respectively.<br>2. The experimental composite containing UMQA-12 and the GIC exhibited similarly low OD_{450} values, indicating less *S. mutans* on the surface after 24 hours compared to the Z250 composite.
Cheng et al. [10]	• Live/Dead biofilm staining<br>• Lactate dehydrogenase-based enzymatic method to measure lactic acid production	• *Streptococci*	1. Four composites – NACP-QADM, NACP-Ag, NACP-QADM-Ag, and a commercial control – were tested over water-aging periods of 1 day, 3, 6, 9, and 12 months.<br>2. All experimental antibacterial composites exhibited relative more dead bacteria, consistent across all aging periods.<br>3. CFU counts for each composite did not significantly change over time.<br>4. Metabolic activity and lactic acid production corresponded to CFU results, with NACP-QADM-Ag exhibiting the lowest acid production.
Makvandi et al. [11]	• Direct contact test	• *Escherichia coli - PTCC 1330*<br>• *Pseudomonas aeruginosa - PTCC 1074*	1. Control resin (1:1 bis-GMA/TEGDMA) showed no inhibition zones, confirming no inherent antimicrobial activity.

		• *Staphylococcus aureus - ATCC 35923*<br>• *Bacillus subtilis - PTCC 1023*<br>• *Streptococcus mutans - PTCC 1683*<br>• *Candida albicans - PTCC 5027*	2. Addition of QABGMA at 5 -15 wt.% produced distinct antibacterial effects, particularly against gram-positive strains.<br>3. All experimental resins exhibited good inhibitory activity against *E. coli*, but none inhibited *P. aeruginosa*.<br>4. QABGMA effectively inhibited *C. albicans*, with antifungal activity higher than commercial antibiotics even at lower concentrations.
Huang et al. [12]	• Live/Dead staining<br>• Gene expression analysis	• *S. mutans - UA159*	1. Adding 10% MAE-HB significantly suppressed bacterial growth; most surface bacteria were dead.<br>2. Antibacterial activity remained stable over time.<br>3. Expression of *gtfB* and *gtfC* genes was reduced in the MAE-HB resin, with *gtfD* showing no significant change.

References

1. Drejka, P.; Chrószcz-Porębska, M.; Kazek-Kęsik, A.; Chladek, G.; Barszczewska-Rybarek, I. Chemical Modification of Dental Dimethacrylate Copolymer with Tetramethylxylylene Diisocyanate-Based Quaternary Ammonium Urethane-Dimethacrylates—Physicochemical, Mechanical, and Antibacterial Properties. *Materials* **2024**, *17*, 298, doi:10.3390/ma17020298.
2. Chrószcz-Porębska, M.; Kazek-Kęsik, A.; Chladek, G.; Barszczewska-Rybarek, I. Novel Mechanically Strong and Antibacterial Dimethacrylate Copolymers Based on Quaternary Ammonium Urethane-Dimethacrylate Analogues. *Dent. Mater.* **2023**, *39*, 659–664, doi:10.1016/j.dental.2023.05.008.
3. Chrószcz-Porębska, M.W.; Barszczewska-Rybarek, I.M.; Kazek-Kęsik, A.; Ślęzak-Prochazka, I. Cytotoxicity and Microbiological Properties of Copolymers Comprising Quaternary Ammonium Urethane-Dimethacrylates with Bisphenol A Glycerolate Dimethacrylate and Triethylene Glycol Dimethacrylate. *Materials* **2023**, *16*, 3855, doi:10.3390/ma16103855.
4. Zhang, L.; Ma, Z.; Wang, R.; Zuo, W.; Zhu, M. Bis-Quaternary Ammonium Betulin-Based Dimethacrylate: Synthesis, Characterization, and Application in Dental Restorative Resins. *Mater. Adv.* **2023**, *4*, 2127–2137, doi:10.1039/D3MA00016H.
5. Fanfoni, L.; Marsich, E.; Turco, G.; Breschi, L.; Cadenaro, M. Development of Di-Methacrylate Quaternary Ammonium Monomers with Antibacterial Activity. *Acta Biomater.* **2021**, *129*, 138–147, doi:10.1016/j.actbio.2021.05.012.

6. Li, S.; Yu, X.; Liu, F.; Deng, F.; He, J. Synthesis of Antibacterial Dimethacrylate Derived from Niacin and Its Application in Preparing Antibacterial Dental Resin System. *J. Mech. Behav. Biomed. Mater.* **2020**, *102*, 103521, doi:10.1016/j.jmbbm.2019.103521.
7. Manouchehri, F.; Sadeghi, B.; Najafi, F.; Mosslemin, M.H.; Niakan, M. Synthesis and Characterization of Novel Polymerizable Bis-Quaternary Ammonium Dimethacrylate Monomers with Antibacterial Activity as an Efficient Adhesive System for Dental Restoration. *Polym. Bull.* **2019**, *76*, 1295–1315, doi:10.1007/s00289-018-2414-y.
8. Wang, Y.; Costin, S.; Zhang, J.; Liao, S.; Wen, Z.T.; Lallier, T.; Yu, Q.; Xu, X. Synthesis, Antibacterial Activity, and Biocompatibility of New Antibacterial Dental Monomers. *Am. J. Dent.* **2018**, *31*, 17B-23B.
9. Huang, Q.; Huang, S.; Liang, X.; Qin, W.; Liu, F.; Lin, Z.; He, J. The Antibacterial, Cytotoxic, and Flexural Properties of a Composite Resin Containing a Quaternary Ammonium Monomer. *J. Prosthet. Dent.* **2018**, *120*, 609–616, doi:10.1016/j.prosdent.2017.12.017.
10. Cheng, L.; Zhang, K.; Zhou, C.-C.; Weir, M.D.; Zhou, X.-D.; Xu, H.H.K. One-Year Water-Ageing of Calcium Phosphate Composite Containing Nano-Silver and Quaternary Ammonium to Inhibit Biofilms. *Int. J. Oral Sci.* **2016**, *8*, 172–181, doi:10.1038/ijos.2016.13.
11. Makvandi, P.; Ghaemy, M.; Mohseni, M. Synthesis and Characterization of Photo-Curable Bis-Quaternary Ammonium Dimethacrylate with Antimicrobial Activity for Dental Restoration Materials. *Eur. Polym. J.* **2016**, *74*, 81–90, doi:10.1016/j.eurpolymj.2015.11.011.
12. Huang, L.; Yu, F.; Sun, X.; Dong, Y.; Lin, P.; Yu, H.; Xiao, Y.; Chai, Z.; Xing, X.; Chen, J. Antibacterial Activity of a Modified Unfilled Resin Containing a Novel Polymerizable Quaternary Ammonium Salt MAE-HB. *Sci. Rep.* **2016**, *6*, 33858, doi:10.1038/srep33858.
